# Prognostic value of plasma adipokine chemerin in patients with coronary artery disease

**DOI:** 10.3389/fcvm.2022.968349

**Published:** 2022-09-08

**Authors:** Bo Wang, Wenxin Kou, Shuya Ji, Rongrong Shen, Hongwei Ji, Jianhui Zhuang, Yifan Zhao, Bo Li, Wenhui Peng, Xuejing Yu, Hailing Li, Yawei Xu

**Affiliations:** Department of Cardiology, Shanghai Tenth People’s Hospital, Tongji University School of Medicine, Shanghai, China

**Keywords:** adipokine, chemerin, coronary artery disease, major adverse cardiac events, prognosis

## Abstract

**Background:**

Adipokine chemerin was proven to be associated with coronary artery disease (CAD), but its prognostic implications in CAD remain unclear.

**Methods:**

This study consists of two parts, one is a basic study and the other is a clinical cohort study. First, we investigated the differential expression of six adipokines in the atherosclerotic mice model compared to mice with milder degrees of atherosclerosis and mice without atherosclerosis using microarray data. We then examined the potential of chemerin as a diagnostic and prognostic indicator in a CAD cohort. A total of 152 patients were enrolled in our study, including 77 patients with angiographically proven CAD and 75 control subjects without cardiovascular disease. Plasma adipokine chemerin levels were measured in all patients, and major adverse cardiovascular events (MACEs) were followed up, including ischemic stroke, non-fatal myocardial infarction, revascularization, and cardiovascular death.

**Results:**

In the aortas of atherosclerotic mice, chemerin expression was up-regulated compared to control mice. The plasma chemerin levels of CAD patients were higher than those of non-CAD patients (128.93 ± 37.06 vs. 109.85 ± 27.47 mmol/L, respectively, *P* < 0.001). High chemerin levels were an independent predictor of CAD (β = 2.702, 95% CI, 1.344–5.431, *P* = 0.001). We followed up with patients for a median duration of 5.5 years (3.9–5.6). The Kaplan–Meier curves showed that patients in the high chemerin group had a significantly higher risk of MACEs than the low chemerin group in patients with CAD (log-rank *P* = 0.003), not with non-CAD (Log-rank *P* = 0.120). Furthermore, Cox multivariate analysis revealed that high chemerin levels were an independent predictor of MACEs (HR 2.267; 95% CI, 1.139–4.515; *P* = 0.020). Finally, the cellular study showed that chemerin is predominantly expressed in PBMC-derived macrophages.

**Conclusion:**

Plasma chemerin levels were increased in the CAD patients, and a high chemerin level increased the risk of MACEs in CAD patients.

## Introduction

CAD is one of the most common health issues, with high morbidity and mortality. Therefore, the early diagnosis and risk stratification of CAD is particularly important. The metabolic syndrome is a risk factor for many diseases, including type 2 diabetes mellitus (DM), insulin resistance, cardiovascular diseases, and liver diseases (1). Obesity plays a critical role in metabolic syndrome (2). Adipocytes distribute throughout the body and are associated with multiple organs, including the heart and vessels (3). Adipokines are bioactive molecules produced by adipose tissue, participating in adipocyte maturation and differentiation, and playing a key role in the integration of systemic metabolism (4). Adipokines like leptin, resistin, visfatin, and chemerin are described as pro-inflammatory markers involved in atherosclerotic plaque progression. Among them, adipokine chemerin is reported to be associated with inflammation, endothelial dysfunction, metabolic disorder, abnormal angiogenesis, vascular smooth muscle cell proliferation, and calcification (5). Chemerin is the agonist of the chemokine-like receptor 1, which is found in dendritic cells, monocytes/macrophages, and adipocytes (6–9).

Previous studies showed that chemerin levels in patients with CAD were significantly increased and positively correlated with the severity of atherosclerosis and macrophage foam cells in aortic plaques (10–12). In this study, we investigated the expression of adiponectin, visfatin, leptin, resistin, and novel adipokines chemerin and vaspin in the whole aortas isolated from mice with or without atherosclerosis and mice with relieved atherosclerosis after taking angiotensin-converting enzyme inhibitor (ACEI) to prevent atherosclerosis development using bioinformatic methods. We also examined the potential of chemerin as a diagnostic and prognostic indicator in a CAD cohort. Given the high chemerin expression in macrophages in human aortic plaques, we performed an *in vitro* study of PBMC-derived macrophages.

## Materials and methods

### Microarray data acquisition and process

The microarray dataset of GSE19286 was retrieved from the GEO database (13). Four- to six-week-old APOE-deficient (APOE^–/–^) mice on a C57BL/6J background and non-transgenic mice without atherosclerosis were used as controls. Age-matched APOE^–/–^ mice were treated for 7 months with the ACEI. At the age of 32–34 weeks, ACEI reduced the development of atherosclerotic plaques in the aorta of APOE^–/–^ mice. In contrast, age-matched APOE^–/–^ mice had a significant plaque load in the aortic arch. The aorta was isolated at this time for microarray gene expression profiling. The original expression matrix was normalized and processed by R. The heatmap was generated for visualization.

### Study population and clinical follow-up design

A total of 152 patients with suspected myocardial ischemia who were hospitalized in Shanghai Tenth People’s Hospital from July 2010 to December 2010 were included in the study. All patients underwent coronary angiography, of which 77 patients were proved to have CAD (diameter stenosis ≥ 50%) and 75 control subjects were without significant CAD (diameter stenosis < 50%). The inclusion criteria and diagnosis criteria were as our previous study reported (14). Subjects with acute myocardial infarction, heart failure, or cardiomyopathies were excluded. Exclusion criteria for all study participants also include acute infection, an acute state of a chronic infectious or inflammatory disease, severe liver or renal disease, neoplasm, and hematologic disorders. All procedures performed on subjects were followed by the Helsinki Declaration of 1975. This study was approved by the Shanghai Tenth Hospital’s Ethics Committee and written informed consent was obtained from all participants.

For the entire cohort of 152 subjects, the primary outcome was a composite of major adverse cardiovascular events (MACEs) at all follow-ups, including ischemic stroke, non-fatal myocardial infarction (NFMI), revascularization, and cardiovascular death. NFMI was defined by a rise of either troponin I > 1.0 ng/mL or troponin T > 0.1 ng/mL with canonical chest pain symptoms and/or characteristic electrocardiographic changes. Revascularization with either percutaneous coronary intervention or coronary artery bypass grafting was confirmed by a review of the medical records. Ischemic stroke was defined as the new onset of neurological deficiency symptoms for at least 24 h with evidence from either magnetic resonance imaging or computed tomography. The follow-up process was a combination of telephone contact and a review of the computerized medical records of clinic visits and hospitalizations. The median follow-up duration was 5.5 years (Q1-Q3 3.9–5.6). Based on the relatively small number of participants, no blanking period was performed.

### Coronary angiography

Coronary angiography was performed by a standard Judkins technique or a radial approach. CAD definition and coronary atherosclerosis severity classification were conducted as our previous study reported (15). The percutaneous coronary intervention was performed according to the 2011 ACCF/AHA/SCAI Guidelines (16). The imaging analyses were conducted by two experienced interventional cardiologists who were blind to the patients’ clinical data.

### Data collection and chemerin measurement

Demographic data, cardiovascular risk factors, and biochemical indicators were collected from each participant. Plasma was collected by centrifuging at 1,000 g for 10 min from each individual after an overnight fast, and then plasma was frozen and stored at −80^°^C until further use. At the end of enrollment of all patients, we examined all plasma samples for chemerin levels in November 2010. Human total adiponectin (R & D Systems, Minneapolis, MN, United States) and chemerin (Wuhan USCN Science Co., Ltd., China) levels in blood plasma were measured by enzyme-linked immunosorbent assays according to the manufacturers’ instructions.

### Cell culture

Human umbilical vein endothelial cells (HUVECs; Cat#8000) were cultured in endothelial cells medium (Cat#1001) supplemented with 5% fetal bovine serum (FBS; Cat#0025), 1% endothelial cell growth supplement (Cat#1052), and 1% penicillin/streptomycin (PS) (Cat#0503). Human aortic smooth muscle cells (HASMCs, Cat#6110) were cultured in smooth muscle cell medium (Cat#1101) supplemented with 2% FBS (Cat#1101), 1% smooth muscle cell growth supplement (Cat#1152), and 1% PS (Cat#0503). THP-1 cells were cultured in RPMI-1640 medium (Gibco, C11875500BT) containing 10% FBS (Gibco, 10099-141) + 1% PS (Gibco, 15140-122). All the cells and reagents aforementioned were purchased from ScienCell, San Diego, CA, United States. After 48 h of culture, the RNA and protein of these three cells were extracted.

Volunteers were recruited among healthy men. We first diluted blood with PBS. Ficoll-Paque PLUS centrifuge was used to isolate peripheral blood mononuclear cells (PBMCs). After centrifugation for 30 min at 400*g, PBMCs were collected from the interphase cloudy layer. PBMCs were suspended in RPMI 1640 supplemented with 10% FBS and 1%PS and were incubated with macrophage colony-stimulating factor (M-CSF) (100 ng/ml, Peprotech) for 7 days to differentiate into monocyte-derived macrophages. All the cells were maintained at 37°C in 5% CO2. After removing the culture medium, we added lipopolysaccharide (LPS) (1 μg/ml, Sigma–Aldrich) and Interferon−γ (IFN−γ) (10 ng/ml, Immunotools), or Interleukin-4 (IL-4) (20 ng/ml, Immunotools) and IL-13 (5 ng/ml; Immunotools) to polarize M0 macrophages toward M1 or M2 phenotype by incubation for 48 h (17).

### RNA isolation and quantitative real-time polymerase chain reaction

Total RNA was extracted using Trizol reagent (Thermo Fisher Scientific). 1 μg of RNA and PrimeScript reverse transcription reagent kit (TaKaRaBio, Shiga, Japan) were used to perform reverse transcription. Real-time qPCR was performed on a LightCycler 96 Real-Time PCR System (F. Hoffmann-La Roche) using FastStart Essential DNAGreen Master assay (F. Hoffmann-La Roche). The 2ΔΔCt method was used for semiquantitative analysis. GAPDH served as an internal standard. Primers were used as follows: chemerin-forward, 5′-GCAGACAAGCTGCCGGA-3′; chemerin-reverse, 5′-AGTTTGATGCAGGCCAGGC-3′; GAPDH-forward, 5′-ACG GATTTGGTCGTATTGGG-3′; GAPDH-reverse, 5′-TGATTTT GGAGGGATCTCGC-3′.

### Western blotting

The protein of cells was extracted using?RIPA buffer (Cell Signaling Technologies, #9806) containing protease inhibitors (Roche Molecular Biochemicals, 04693159001). Protein concentrations were determined using a bicinchoninic acid protein assay. Proteins were separated by SDS–PAGE, transferred to polyvinylidene fluoride (PVDF) membranes and incubated overnight at 4^°^C with primary antibodies including anti-Chemerin (Abclonal, A6963, 1:1000), anti-GAPDH (Proteintech, 60004-1-Ig, 1:10,000). Primary antibodies were then incubated with secondary antibodies for one hour, and bends were visualized using chemiluminescence (ECL, TANON, China) and viewed under Amersham Imager 600 system (GE Healthcare, United States).

### Statistical analysis

Categorical variables are presented as frequencies (percentages), and continuous variables as means and standard deviations or medians with interquartile ranges (IQR) according to distribution based on the Kolmogorov-Smirnov test and visual inspection of Q-Q plots. Comparisons between groups were analyzed with unpaired *t*-tests or a non-parametric Mann-Whitney *U* test when appropriate. The Chi-square test or Fisher exact test was employed for categorical variables. Subsequently, analysis was undertaken through univariate and multivariate logistic regression to assess a set of independent variable predictors of CAD presence. MACE-free survival rates were calculated using the Kaplan-Meier analysis and compared by the log-rank test. To evaluate predictors of the clinical endpoints, Cox regression models were used to derive the adjusted hazard ratio (HR) for MACE events. The receiver operating characteristic curve (ROC) was used to analyze the accuracy and optimum cut-off value of chemerin for clinical MACE prediction, and the Youden index was calculated at the point where the sensitivity and specificity sum was highest. A two-sided *P* ≤ 0.05 was regarded as significant. All analyses were done with SPSS (version 22.0, SPSS Inc., Chicago, IL, United States). Figures were performed using Graphpad Software (version Graphpad Prism 9.0.0).

## Results

### Investigation of adipokine expression in aorta from microarray data

**FIGURE 1:**
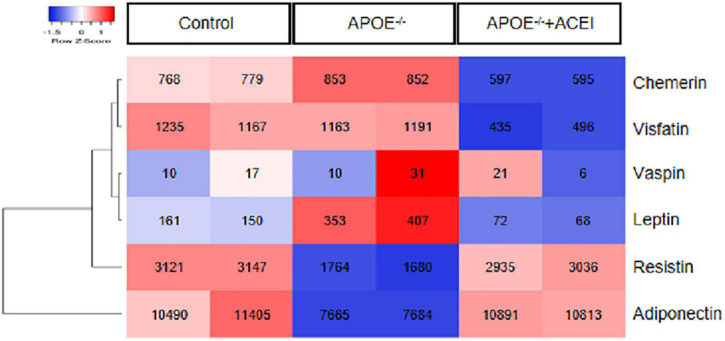
The expression of genes encoding adipokines. The heatmap depicts the down-regulated (blue) and up-regulated (red) expression of the adipokines, including adiponectin, visfatin, leptin, resistin, chemerin, and vaspin in aortas from dataset GSE19286 including APOE^– /–^ mice in the presence or absence of ACEI and control mice (*n* = 2 per condition). ACEI, angiotensin-converting enzyme inhibitor.

We chose adipokines like adiponectin, visfatin, leptin, resistin, chemerin, and vaspin to investigate the crucial adipokines that may play roles in atherosclerosis. As represented in Figure 1, APOE^–/–^ mice exhibited lower expression of resistin and adiponectin and higher expression of leptin and chemerin in aortas compared to control mice, which could be restored after ACEI treatment. This finding indicates that resistin and adiponectin have a negative correlation with atherogenesis. On the contrary, leptin and chemerin were demonstrated to be positively correlated with atherosclerotic progression. Considering the robust change and high intensity of chemerin, we then investigated its correlation with our clinical data.

### Characteristics of the study population

Table 1 shows baseline characteristics in CAD and non-CAD groups. All 152 patients underwent angiography. Seventy-seven patients with CAD, including stable angina pectoris (*n* = 54) and unstable angina pectoris (*n* = 23), and 75 patients without CAD were included in this study. 85% were males with a mean age of 65.0 ± 11.4 years, mean chemerin level was 119.52 ± 33.95 ng/ml. The CAD group showed a significantly higher chemerin level compared to the non-CAD group (128.93 ± 37.06 vs. 109.85 ± 27.47 mmol/L, respectively, *P* < 0.001). Patients with CAD had a higher proportion of diabetes mellitus history (*P* = 0.031) and higher usage of statins (*P* = 0.001). High sensitivity C-reactive protein (hsCRP) levels were higher in the CAD group (median, 5.2 mmol/L, IQR, 3.3–11.7) than in the non-CAD group (median: 3.3 mmol/L; IQR: 3.3–5.2) (*P* = 0.001). The median creatinine was higher in the CAD group than that of the non-CAD group (81.0 [IQR: 71.5–95.5] vs. 75.0 mg/L [IQR: 63.0–87.9], respectively, *P* = 0.043). The mean uric acid level in the CAD group was higher (368.30 ± 100.12 vs. 334.65 ± 98.42 mg/L, respectively, *P* = 0.038). There were no differences between the two groups in other baseline characteristics, laboratory examinations, and discharge medications. Notably, of 152 patients, 22 were on ACEI or angiotensin receptor blocker (ARB) treatment, 11 each in CAD and non-CAD groups (*P* = 0.947).

**TABLE 1:** Baseline characteristics in CAD/non-CAD groups.

Variables	Total *n* = 152	Non-CAD *n* = 75	CAD *n* = 77	*P*-value
**Demographics**				
Age, years	65.0 ± 11.4	64.8 ± 12.8	65.2 ± 10.0	0.854
Male, *n* (%)	85 (55.9)	38 (50.7)	47 (61.0)	0.198
BMI, kg/m^2^	24.56 ± 3.49	24.47 ± 3.33	24.65 ± 3.66	0.751
SBP, mmHg	143.73 ± 22.64	143.37 ± 23.47	144.08 ± 21.94	0.849
DBP, mmHg	81.76 ± 12.96	80.64 ± 13.38	82.847 ± 12.53	0.296
**Comorbidities, *n* (%)**				
DM	38 (25.0)	13 (17.3)	25 (32.5)	0.031
Smoking	32 (21.1)	12 (16.0)	20 (26.0)	0.132
Hypertension	98 (64.5)	46 (61.3)	52 (67.5)	0.425
Hyperlipidemia	16 (10.5)	7 (9.3)	9 (11.7)	0.636
Stroke	20 (13.2)	10 (13.3)	10 (13.0)	0.950
**Medications, *n* (%)**				
Statin	23 (15.1)	4 (5.3)	19 (24.7)	0.001
ACEI/ARB	22 (14.5)	11 (14.7)	11 (14.3)	0.947
CCB	27 (17.8)	12 (16.0)	15 (19.5)	0.575
β-Blocker	8 (5.3)	5 (6.7)	3 (3.9)	0.492
**Laboratory data**				
LVEF, %	64.13 ± 8.31	64.72 ± 7.02	63.56 ± 9.41	0.391
Pro-BNP	124 (50–299)	124 (47–295)	124 (57–303)	0.666
TC, mmol	4.53 ± 1.08	4.64 ± 1.02	4.42 ± 1.14	0.217
TG, mmol/L	1.47 (1.07–1.99)	1.36 (1.02–1.91)	1.55 (1.14–2.02)	0.191
HDL-C, mmol/L	1.13 ± 0.30	1.16 ± 0.29	1.09 ± 0.30	0.137
LDL-C, mmol/L	2.53 (1.98–2.98)	2.6 (2.10–2.98)	2.40 (1.94–2.96)	0.122
FPG, mmol/L	5.2 (4.8–6.1)	5.2 (4.7–5.7)	5.3 (4.8–6.4)	0.126
HbA1c, mmol/L	6.0 (5.5–6.3)	6.0 (5.4–6.2)	6.00 (5.6–6.8)	0.141
HsCRP, mg/L	3.5 (3.3–7.9)	3.3 (3.3–5.2)	5.2 (3.3–11.7)	0.001
AST, U/L	18 (15–24)	18 (15–22)	20 (17–25)	0.101
ALT, U/L	18 (12–26)	16 (12–23)	19 (14–26)	0.165
BUN, mmol/L	5.92 ± 2.12	5.79 ± 2.35	6.04 ± 1.87	0.461
Creatinine, mg/L	78.5 (67.0–91.0)	75.0 (63.0–87.9)	81.0 (71.5–95.5)	0.043
Uric acid, mg/L	351.69 ± 100.39	334.65 ± 98.42	368.30 ± 100.12	0.038
Adiponectin, mg/mL	11.40 ± 8.31	11.15 ± 7.25	11.64 ± 9.27	0.717
Chemerin, ng/mL	119.52 ± 33.95	109.85 ± 27.47	128.93 ± 37.06	<0.001

Values are mean ± standard deviation, median (interquartile range), or n (%). CAD, coronary artery disease; BMI, body mass index; SBP, systolic blood pressure; DBP, diastolic blood pressure; n, number of patients; DM, diabetes mellitus; ACEI/ARB, angiotensin-converting enzyme inhibitor and angiotensin receptor blocker; CCB, calcium channel blocker; LVEF, left ventricular ejection fraction; Pro-BNP, pro-brain natriuretic peptide; TC, total cholesterol; TG: Triglycerides; LDL-C, low-density lipoprotein cholesterol; HDL-C, high-density lipoprotein cholesterol; FPG, fasting plasma glucose; HbA1c, Hemoglobin A1c; AST, aspartate transaminase; BUN, blood urine nitrogen; hsCRP, high sensitivity C-reactive protein.

### Baseline of chemerin grouping and the correlation between chemerin and coronary artery disease

The patients were classified into two groups based on a chemerin cut-off value of 109.27 ng/ml. The cut-off of 109.27 ng/ml for chemerin was derived from ROC analysis based on MACE prediction (Supplementary Figure 1). Supplementary Table 1 shows the baseline characteristics of the two groups. 85 patients (55.9%) were in the high chemerin group (chemerin ≥ 109.27 ng/ml), while 67 patients (44.1%) were in the low chemerin group. The proportion of patients with CAD in the high chemerin group was higher than that in the low chemerin group (62.4 vs. 35.8%, respectively, *p* = 0.001). The two groups had similar numbers of patients with hypertension, while the high chemerin group had higher usage of calcium channel blockers (10.4 vs. 23.5%, respectively, *p* = 0.038). Moreover, the high chemerin group had significantly more diabetic patients than the low chemerin group (32.9 vs. 14.9%, respectively, *p* = 0.011), and the median fasting blood glucose level was also higher (5.1 [IQR: 4.7–5.7] vs. 5.4 mmol/L [IQR: 4.9–6.5], respectively, *P* = 0.008).

Supplementary Table 2 shows binary logistic regression analysis for correlative factors to predict CAD. Univariate and multivariate analysis shows the high chemerin level was an independent predictor of CAD (β = 2.702, 95% CI, 1.344–5.431, *P* = 0.001) after adjusting for age, sex, diabetes, hypertension, hyperlipidemia, obesity, and smoking.

### Follow-ups: High plasma chemerin level predicted major adverse cardiovascular events in patients with coronary artery disease

To explore the role of chemerin in the prognosis of CAD, we followed up patients for 5.5 years (3.9–5.6). The incidence of the following MACEs among the high/low groups for the initial 152 patients was presented in Table 2. 56 patients were documented with a MACE (56/152, 36.8%). Of the 56 MACEs, 15 were in the non-CAD group and 41 in the CAD group. In the high/low chemerin subgroup analysis, the high chemerin group had a higher prevalence of MACEs than the low chemerin group in overall patients (*P* < 0.001) and CAD patients (*P* = 0.003), while there was no significant difference in non-CAD patients (*p* = 0.120). Notably, among all endpoint events, revascularization (*P* = 0.015) and stroke (*P* = 0.008) had a significant difference in all patients, whereas among CAD patients, only the incidence of stroke differed (*P* = 0.044).

**TABLE 2:** Comparison of MACEs among high/low chemerin groups.

Variables	Low chemerin (*n* = 67)	Total high chemerin (*n* = 85)	*P*-value	Low chemerin (*n* = 43)	Non-CAD high chemerin (*n* = 32)	*P*-value	Low chemerin (*n* = 24)	CAD high chemerin (*n* = 53)	*P*-value
MACEs	12	44	<0.001	6	9	0.120	6	35	0.003
Cardiac death	1	6	0.077	0	2	0.101	1	4	0.466
NFMI	5	12	0.141	3	2	0.945	2	10	0.193
Stroke	4	14	0.008	2	5	0.087	0	7	0.044
Revascularization	2	12	0.015	1	0	0.474	3	14	0.081

Data are n. MACE, major adverse cardiovascular events; NFMI, non-fatal myocardial infarction; CAD, coronary artery disease; P-values were derived using the log-rank test.

**FIGURE 2:**
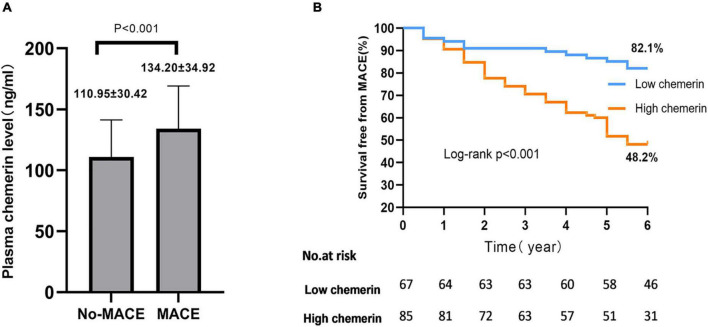
Plasma chemerin concentrations and MACEs-free of high/low chemerin groups in all patients. **(A)** Plasma chemerin concentrations were higher in the MACE group compared to the non-MACEs group. **(B)** Kaplan-Meier curves for freedom from MACEs stratified by chemerin group. MACE, major adverse cardiovascular events.

**FIGURE 3:**
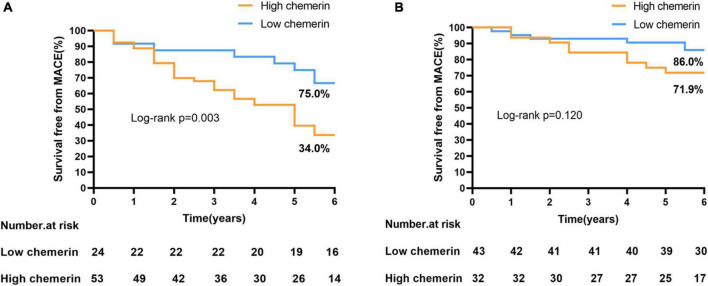
Incidence of MACEs-free in patients with or without CAD. **(A)** High/low chemerin in patients with CAD. **(B)** High/low chemerin in patients with non-CAD. MACEs, major adverse cardiovascular events; CAD, coronary artery disease.

Plasma chemerin levels in the MACEs group were significantly higher than those of the non-MACEs group (134.20 ± 34.92 vs. 110.95 ± 30.42 g/mL, *p* < 0.001) (Figure 2A). The Kaplan-Meier curves displayed the relationship between the chemerin group and survival free of total MACEs, which showed a significantly increased risk of MACE in patients with a high chemerin group (log-rank *P* < 0.001) (Figure 2B). However, Figure 3 shows the survival rate in patients with high and low chemerin in patients with or without CAD. In patients with CAD, the incidence of high chemerin MACEs was 34.0% compared to 75.0% in the low chemerin group (Figure 3A). In contrast, in the non-CAD group, there was no significant difference in MACEs-free between the two groups (Figure 3B). There was compelling evidence for the link between chemerin and the prognosis of patients with CAD. Furthermore, ROC curves confirmed that chemerin could be a predictor of cardiovascular events [area under the curve (AUC) = 0.706, *P* = 0.001] (Supplementary Figure 1).

Table 3 shows the result of the Cox proportional hazard analysis. Factors that were univariate predictors with *P* < 0.20 were considered covariates for the multivariable models. The multivariate analysis revealed that the high chemerin levels were a significant independent predictor of MACE (HR 2.267; 95% CI, 1.139–4.515, *P* = 0.020). In addition, CAD (HR, 2.402; 95% CI, 1.272–4.536, *P* = 0.007), male (HR, 1.805; 95% CI, 1.016–3.207, *P* = 0.044), and creatinine (HR, 1.009; 95% CI, 1.001–1.016, *P* = 0.029) were the other multivariate predictors associated with higher risk for MACEs.

**TABLE 3:** Univariable and multivariable predictors of MACEs (*P* < 0.2).

Factors	Hazard risk	95% CI	*P*-value
**Univariable predictor**			
Male	1.507	0.891–2.547	0.126
DM	2.220	1.297–3.801	0.004
Stroke	1.655	0.856–3.201	0.134
Creatine	1.009	1.003–1.016	0.005
Pro-BNP	1.000	1.000–1.001	0.013
High chemerin	3.672	1.936–6.967	<0.001
HsCRP	1.031	0.984–1.081	0.197
CAD	3.327	1.790–5.853	<0.001
**Multivariate predictor**			
Male	1.805	1.016–3.207	0.044
Creatine	1.009	1.001–1.016	0.029
High chemerin	2.402	1.272–4.536	0.020
CAD	2.402	1.272–4.536	0.007

CI, confidence interval; others are with Tables 1, 2.

### Chemerin was expressed in macrophages

**FIGURE 4:**
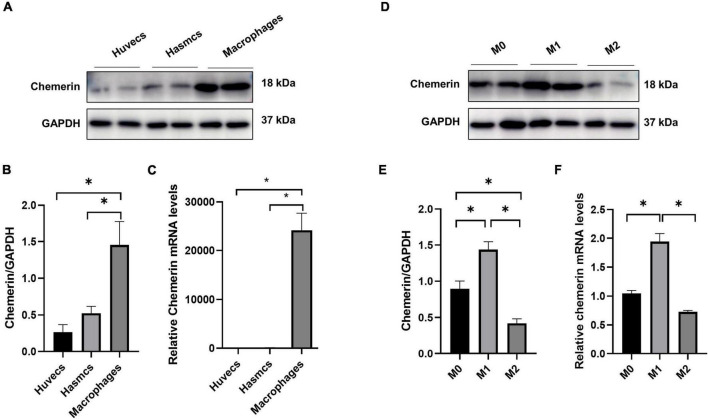
High expression of chemerin in M1 macrophages. **(A–C)** Chemerin mRNA and protein levels were higher in macrophages than in HASMCs and HUVECs. **(D–F)** Higher chemerin mRNA and protein levels were displayed in M1 than in M2 macrophages. Statistically significant *p*-values were marked by *p < 0.05. HUVECs, human umbilical vein endothelial cells; HASMCs, human aortic smooth muscle cells.

It is well established that endothelial cells, smooth muscle cells, and macrophages are involved in the development of AS (18), so we compared the expression level of chemerin in HUVECs, HASMCs, and PBMC-derived macrophages. It was shown that chemerin mRNA and protein levels were more abundant in macrophages than in HASMCs and HUVECs (Figures 4A–C), suggesting chemerin might be released by macrophages and participate in atherogenesis. Macrophages can be typically divided into two classes pro-inflammatory M1 and anti-inflammatory M2 macrophages (19). It was detected that the level of chemerin was much higher in M1 than in M2 macrophages (Figures 4D–F).

## Discussion

The main findings of the current study were as follows. First, in a mouse model, gene expression of chemerin was positively correlated with the severity of atherosclerosis by bioinformatic methods. Second, in CAD patients, the chemerin level was higher than that in non-CAD patients, and high chemerin levels were independently associated with CAD. Third, high chemerin was a significant and independent predictor of MACEs in CAD patients. Fourth, M1 macrophages were responsible for the majority of chemerin expression.

Previous studies have shown that 8 weeks after APOE knockout in mice, aortic plaques formed and aortic chemerin levels increased (20). In this study, we investigated the gene expressions of six adipokines in the aortas of APOE^–/–^ mice in the presence or absence of ACEI treatment and control mice using microarray. The AS degree was inversely connected with resistin and adiponectin, while leptin and chemerin were positively correlated.

We found that chemerin was higher in plasma from CAD patients than in non-CAD patients, which is consistent with recent research that revealed a link between chemerin levels and cardiovascular disease (7). Leiherer et al. first suggested high chemerin levels were associated with predictive of cardiovascular events and renal impairment (21). Besides, serum chemerin concentrations are correlated with atrial fibrillation and atrial remodeling. Atrial fibrillation patients’ chemerin concentrations were higher than healthy controls (22). Increased chemerin levels are also reported to be positively linked to dilated cardiomyopathy (23). More studies were conducted to investigate the role of chemerin in CAD severity. The results illustrated that chemerin might have diagnostic potential in atherosclerosis, and the level of chemerin could predict the severity of CAD.

Atherosclerosis is a chronic inflammatory disease in the arteries that may cause cardiovascular death, ischemic stroke, NFMI, and revascularization, collectively called MACEs. Chemerin was quite distinct between CAD and non-CAD groups. Moreover, for the first time, we illustrated that increased baseline plasma chemerin levels might predict the future occurrence of MACEs in the Chinese population. Interestingly, the occurrence of MACEs was notably lower in the low chemerin group than in the high chemerin group at follow-ups, especially in terms of NFMI, revascularization, and stroke. However, in the CAD and non-CAD groupings, we found that increased chemerin had a higher risk of MACEs in CAD patients, while there was no difference in non-CAD patients. Thus, chemerin could be a potential biomarker for predicting the occurrence of cardiovascular diseases.

We also observed that there is a high proportion of DM patients and high usage of statins in patients with CAD, and the levels of creatinine and uric acid were also higher than those of non-CAD. These traditional high metabolic determinants and indicators have once again been shown to be related to CAD, which also implies that adipokine chemerin may play a role through metabolism. In addition, we found that hsCRP was elevated in patients with CAD. Previous studies have shown that hsCRP was associated with the incidence of cardiovascular events and was involved in atherosclerosis (24), which suggests that chemerin may participate in atherosclerosis through inflammation.

Previous studies have shown that renin-angiotensin system antagonists could decrease cardiovascular events and mortality in stable CAD patients (25, 26), and in the present study, the degree of atherosclerosis was significantly suppressed in mice after 7 months of ACEI administration. In addition, there was a study that showed expression levels of RARRES2, the coding gene of chemerin, and ACEI were independently correlated with the severity of coronary artery disease (CAD) and insulin resistance (27), which suggested there might be a potential association between ACEI and chemerin in patients. However, in our study, we measured chemerin levels in plasma samples from patients during their hospitalization, whereas we collected medication for patients taking ACEI therapy at discharge, which did not reflect the effect of ACEI on chemerin levels. Therefore, we could not draw conclusive results between ACEI and chemerin in patients. Moreover, the amounts of patients on ACEI treatment were relatively low in both groups, and there may be some bias. The relationship between ACEI and chemerin in patients remains to be investigated by further randomized controlled trials.

Although significantly high circulation chemerin concentrations were found in CAD patients (28), the role of chemerin in cardiovascular pathogenesis and the molecular network of chemerin regulation remained largely unknown. Atherogenic processes involve several kinds of cells, including the inflammation and mechanotransduction of endothelial cells (ECs), activation of lesion macrophages, and phenotypic switching of smooth muscle cells (SMCs) (18). Chemerin may affect cardiovascular diseases through the modulation of these cells. It is reported that high levels of chemerin are independently associated with ECs activation (29). Chemerin can alter endothelial adhesion levels and nitric oxide synthase (NOS) expression levels (30). Besides, the increased chemerin expression and activation can enhance the release of NO in vascular ECs, promoting endothelial regeneration. Moreover, chemerin is associated with an increase in phagocytic properties, while the phagocytic capacity of cells is related to the generation of reactive oxygen species and the activation of the AMPK pathway (31, 32). In inflammatory responses, chemerin could attract dendritic cells and macrophages through its receptors ChemR23 (33). Some studies also show chemerin can promote arterial contraction by primary chemerin receptor ChemR23 on SMCs. The activation of ChemR23 promotes the binding of the intracellular calcium ion and calmodulin (34, 35). The chemerin/chemR23 axis is a complex network involved in the regulation of immune responses to the onset as well as resolution of inflammation (36–38). In the present study, we examined chemerin expression in HUVECs, HASMCs, and macrophages. We found that the levels of chemerin in macrophages were much higher than that in HUVECs and HASMCs. When M0 macrophages transform into M1 macrophages, dramatically increased chemerin mRNA was detected, while chemerin expression in M2 macrophages was slightly decreased. The results suggest that the release of chemerin by macrophages increases under inflammatory conditions and might influence the function of other cells to promote atherogenesis. However, a wild mouse aortic genome analysis showed that chemerin is not only expressed in macrophages but also highly expressed in vascular smooth muscle cells and fibroblasts (39). Therefore, the effect of chemerin on macrophages and its mechanism remains to be further investigated. Despite some limitations, our study still has significant implications for the origin of chemerin.

Of note, we found that the level of chemerin was lower in MACEs-free patients. Thus, we propose using chemerin as a novel biomarker for the early diagnosis and prognosis of cardiovascular diseases. Our research may explain the reason why plasma chemerin in CAD patients is significantly increased. To explore the mechanism, translational studies should be strongly encouraged in rodent models of cardiovascular and/or cardiometabolic disease. New drugs for CAD treatment will be developed to target chemerin itself or its receptor in the future.

There were several limitations to our study. At first, the sample size is small, it requires larger cohorts of patients with CAD and control individuals to extensively evaluate chemerin as a potential biomarker. Secondly, since this is not a cross-sectional cohort study, biases in data analysis may exist, which limit the establishment of a cause-effect relationship between chemerin and CAD. Thirdly, the clinical medication data of patients are not comprehensive, such as the use of anti-platelet drugs. Therefore, there is a lack of research on the effect of medication interventions on the chemerin level. In addition, the specific mechanism of chemerin in macrophages underlying AS is still unclear, which needs to be further clarified.

## Conclusion

Our findings showed that plasma chemerin levels were higher in CAD patients, and elevated chemerin level was an independent predictor of the occurrence of MACEs in CAD patients. In addition, the cellular study showed that chemerin is predominantly expressed in PBMC-derived macrophages.

## Data availability statement

The original contributions presented in this study are included in the article/Supplementary material, further inquiries can be directed to the corresponding author/s.

## Ethics statement

The studies involving human participants were reviewed and approved by the Shanghai Tenth Hospital’s Ethics Committee. The patients/participants provided their written informed consent to participate in this study.

## Author contributions

RS, HJ, JZ, and HL: conceptualization. BW and YZ: data curation. XY: formal analysis. YX: funding acquisition. SJ and HJ: investigation. HL: methodology and resources. WP and HL: project administration. XY and YX: supervision. WK and BL: visualization. BL: supplementary cell experiment. BW and WK: writing – original draft, review, and editing. All authors have read and agreed to the published version of the manuscript.

## Abbreviations

CAD: coronary artery disease
MACE: major adverse cardiac event
AS: atherosclerosis
NFMI: non-fatal myocardial infarction
REVAS: revascularization
DM: diabetes mellitus
ARB: angiotensin receptor blocker
ACEI: angiotensin-converting enzyme inhibitor
SD: standard deviation
SBP: systolic blood pressure
DBP: diastolic blood pressure
BMI: body mass index
HsCRP: high sensitivity C-reactive protein
FPG: Fasting plasma glucose
TC: total cholesterol
TG: triglyceride
LDL-C: low-density lipoprotein cholesterol
HDL-C: high-density lipoprotein cholesterol
Pro-BNP: pro-brain natriuretic peptide
LVEF: left ventricular ejection fraction
ROC: receiver operating characteristic
AUC: area under the curve
HUVEC: Human umbilical vein endothelial cell
SMC: smooth muscle cell
PUMC: peripheral blood mononuclear cell
CCB: calcium channel blocker
PCR: polymerase chain reaction
HR: hazard ratio
CI: confidence interval.
