# Supplementary material for: Prognostic value of plasma adipokine chemerin in patients with coronary artery disease
Source: Front Cardiovasc Med. 2022 Sep 8;9:968349. doi: 10.3389/fcvm.2022.968349 (PMC9493186; doi:10.3389/fcvm.2022.968349)
Supplement: Supplementary file 1 [file Data_Sheet_1.pdf]

Supplementary Material

Supplementary Tables and Figures

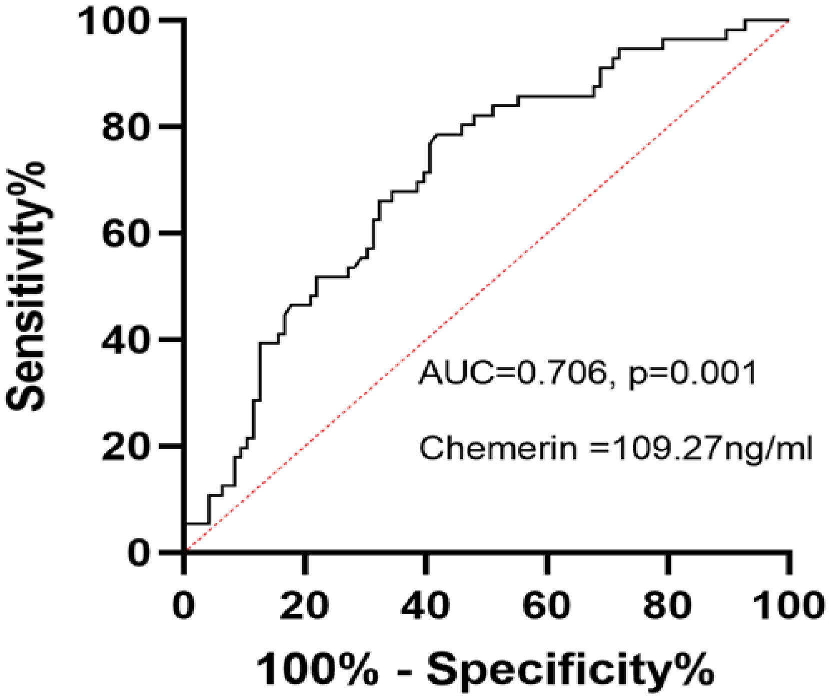

Supplementary Figure 1. The receiver-operating characteristic curve of chemerin to predict major adverse cardiovascular events. AUC, area under the curve.

Supplementary Table 1. Baseline characteristics of patients with high or low chemerin levels.

Variable	Low chemerin n = 67	High chemerin n = 85	P-value
**Demographics**			
Age, years	63.24 ± 11.32	66.39 ± 11.36	0.086
Male, n (%)	37(44)	39(58)	0.102
BMI, kg/m2	24.54 ± 3.13	24.58 ± 3.77	0.948
SBP, mmHg	143.37 ± 23.47	144.08 ± 21.94	0.849
DBP, mmHg	80.64 ± 13.38	82.847 ± 12.53	0.296
**Comorbidities, n (%)**			
CAD	27(40.3)	48(56.5)	0.001
Diabetes mellitus	10(14.9)	28(32.9)	0.011
Smoking	14(20.9)	18(21.2)	0.966
Hypertension	38(56.7)	60(70.6)	0.076
Hyperlipidemia	6(9.0)	10(11.8)	0.575
Stroke	7(10.4)	13(15.3)	0.380
**Medications, n (%)**			
Statin	7(10.4)	16(18.8)	0.153
ACEI / ARB	9(13.4)	13(15.3)	0.746
CCB	7(10.4)	20(23.5)	0.036
β-Blocker	4(6)	4(4.7)	0.729
**Laboratory data**			
LVEF, %	64.36 ± 8.43	63.95 ± 8.26	0.766
Pro-BNP	111(46.7 - 233)	140(52 - 416)	0.068
TC, mmol	4.52 ± 1.01	4.54 ± 1.14	0.897
TG, mmol/L	1.33(1.02 - 1.80)	1.57(1.10 - 2.24)	0.053
HDL-C, mmol/L	1.17 ± 0.28	1.10 ± 0.31	0.154
LDL-C, mmol/L	2.59(1.90 - 2.86)	2.47(2.11 - 3.05)	0.815
FPG, mmol/L	5.1(4.7 - 5.7)	5.4(4.9 - 6.5)	0.008
HbA1c, mmol/L	5.9(5.5 - 6.3)	6.0(5.5 - 6.6)	0.433
HsCRP, mg/L	3.4(3.3 - 6.9)	3.5(3.3 - 8.1)	0.463
AST, U/L	18(14 - 23)	19 (16 - 25)	0.392
ALT, U/L	18(11 - 26)	18(14 - 26)	0.493
BUN, mmol/L	5.67 ± 1.78	6.11 ± 2.34	0.201
Creatinine, mg/L	76.0(64.0 - 87.9)	79.0(71.0 - 92.5)	0.214
Uric acid, mg/L	349.02 ± 97.18	353.80 ± 103.37	0.772
Adiponectin, mg/mL	11.50 ± 7.47	11.17 ± 9.25	0.249
Chemerin, ng/mL	90.39 ± 12.45	142.48 ± 27.21	0.001

Values are mean ± SD, median (interquartile range), or n (%). CAD, coronary artery disease; BMI, body mass index; SBP, systolic blood pressure; DBP, diastolic blood pressure; n, number of patients; ACEI/ARB, angiotensin-converting enzyme inhibitor and angiotensin receptor block agent; CCB,

calcium channel blocker; LVEF, left ventricular ejection fraction; Pro-BNP, pro-brain natriuretic peptide; TC, total cholesterol; TG: Triglycerides; LDL-C, low-density lipoprotein cholesterol; HDL-C, high-density lipoprotein cholesterol; FPG, fasting plasma glucose; HbA1c: Hemoglobin A1c; AST, aspartate transaminase; BUN, blood urine nitrogen; hsCRP, high sensitivity CRP.

Supplementary Table 2. Results of binary logistic regression analysis for correlative factors with CAD at baseline.

Variables	Univariable			Multivariable		
	Exp(β)	95% CI	P-Value	Exp(β)	95% CI	P-value
Age, y	1.003	0.975 - 1.031	0.853	0.996	0.964 - 1.029	0.799
Male	0.656	0.344 - 1.248	0.199	0.605	0.275 - 1.331	0.212
Hypertension	1.311	0.674 - 2.552	0.425	1.085	0.515 - 2.283	0.830
DM	2.293	1.067 - 4.927	0.033	2.115	0.889 - 5.035	0.091
Hyperlipidemia	1.286	0.453 - 3.650	0.637	1.396	0.454 - 4.291	0.560
Smoking	1.842	0.827 - 4.101	0.135	1.545	0.592 - 4.035	0.374
Obesity	0.871	0.445 - 1.706	0.688	0.775	0.359 - 1.673	0.517
High chemerin	2.967	1.526 - 5.769	0.001	2.702	1.344 - 5.431	0.005

CI, confidence interval; CAD, coronary artery disease; DM: Diabetes mellitus; Obesity is defined as BMI ≥ 25 kg/m^2.

Sample Size Calculation

We projected a 5-year major adverse cardiovascular events of 19% in patients with coronary artery diseases and 3% in patients with no-obstructive coronary artery diseases according to the previous reports. Sample size estimates are predicated on the following assumptions:

- a trial duration of 5.5 years;
- 6 months to complete patient enrollment;
- average follow-up duration of 5.5 months;
- two-sided tests of significance at an alpha = 0.05;
- power of test 90%;

The total sample size was calculated to be 104, with 52 in the CAD group and 52 in the control group. Through six months of enrollment, we finally included 152 participants, 77 in the CAD group and 75 in the control group.
